# Effects of neighbourhood income on reported body mass index: an eight year longitudinal study of Canadian children

**DOI:** 10.1186/1471-2458-8-16

**Published:** 2008-01-14

**Authors:** Lisa N Oliver, Michael V Hayes

**Affiliations:** 1 Department of Geography, Simon Fraser University, Burnaby, BC, Canada; 2 Faculty of Health Sciences, Simon Fraser University, Burnaby, BC, Canada

## Abstract

**Background:**

This study investigates the effects of neighbourhood income on children's Body Mass Index (BMI) from childhood (ages 2–3) to early adolescence (ages 10–11) using longitudinal data.

**Methods:**

Five cycles of data from the Canadian National Longitudinal Survey of Children and Youth are analyzed for a sub-sample of children (n = 2152) aged 2–3 at baseline (1994) and assessed at two year intervals to 2002. Body mass index percentiles are based on height/weight estimates reported by proxy respondents (child's person most knowledgeable). Family and neighbourhood factors were assessed at baseline. The prevalence of neighbourhood low income was obtained from the 1996 Census and divided into three categories from 'most poor' to 'least poor'. Longitudinal modelling techniques were applied to the data.

**Results:**

After controlling for individual/family factors (age, sex, income, education, family structure) living in the 'most poor' neighbourhood was associated with increasing BMI percentile (1.46, 95% CI 0.16 to 2.75) over time compared to a 'middle' income neighbourhood. Living in an urban (vs. rural) neighbourhood was associated with a decreased BMI percentile (-3.57, 95% CI -6.38 to -0.76) across all time periods.

**Conclusion:**

These findings provide evidence that effects of neighbourhood disadvantage on children's BMI occur between childhood and early adolescence and suggest that policies should target the conditions of childhood, including the neighbourhood environment.

## Background

The prevalence of childhood obesity and overweight has increased dramatically over the last three decades in most developed nations [1]. The prevalence of overweight among Canadian children has doubled from 13% in 1977/8 to 26% in 2004 among 6-to 11-year olds [2]. These increases are troubling because obesity in childhood persists into adulthood and may be linked to poor long-term health outcomes [3,4]. Cross-sectional research from developed nations has demonstrated an association between neighbourhood disadvantage and an increased prevalence of obesity and overweight among children and youth [5-8]. Longitudinal studies are needed to establish causation and to examine the influence of neighbourhood disadvantage on children's body weights over time. Longitudinal studies of neighbourhood effects on children's health that include both individual and neighbourhood characteristics are rare [9].

Disparities in obesity may emerge between early childhood and adolescence [10]. Neighbourhoods provide a social and physical context which structures opportunities for children to engage in behaviours that promote or inhibit weight gain [11,12]. Residing in a disadvantaged neighbourhood may promote weight gain through access to a less healthy food supply, limited access to recreation facilities and increased safety concerns [6,11,13-15]. Neighbourhood factors may be more influential as children age and have more freedom to access the neighbourhood [16].

Several cross-sectional studies have found that relationships between neighbourhood deprivation and overweight are stronger for older children than younger children [17-19]. Collectively these findings suggest that disparities between overweight and neighbourhood deprivation may emerge between early childhood and adolescence.

The primary goal of this study was to assess the impact of the neighbourhood environment on children's BMI from early childhood to adolescence while controlling for family factors. It was hypothesised that from early childhood to adolescence there would be increasing disparity in body weight by neighbourhood income as neighbourhood factors become more influential and children are exposed to such environments over time.

## Methods

### Sample

Data used were from the Canadian National Longitudinal Survey of Children and Youth (NLSCY), a representative survey of Canadian children aged 0–11 beginning in 1994. Children are assessed bi-annually until age 25 and data used are from the first 5 cycles. The NLSCY uses a complex survey design based on the Statistics Canada Labour Force Survey and a full description of the data is available elsewhere [20]. The initial cohort included 2,916 children aged 2–3 in 1994 (Cycle 1) and we use data for 2,229 respondents aged 10–11 in 2002 (Cycle 5) representing 76.4% of the original cohort.

### Measures

#### Individual

In the NLSCY, children's heights and weights were reported by the person most knowledgeable (PMK) for ages 2–11 and self-report for older children. In Cycle 1 the PMK was the mother in 90% of cases and the father in 8% of cases. We limited our sample to a cohort of children aged 2–3 in Cycle 1. At Cycle 5 these children are aged 10–11 and heights and weights are reported by the PMK across all cycles. All individual and family variables were obtained from Cycle 1. Heights and weights were used to calculate Body Mass Index (BMI – kilograms (kg)/meters (m)^2^), which was then used to derive the outcome variable. We assess BMI percentile using age and sex specific values from the US Centre for Disease Control (CDC)[21] which have been applied to Canadian data [22,23]. This method assigns percentile values to age and sex adjusted BMI z-scores at one month intervals. Based on the CDC cut-offs we identify those with a BMI percentile ≥ 85^th ^as overweight. The CDC categorization refers to the 85^th ^percentile as 'at risk of overweight' and the 95^th ^percentile as 'overweight'. In this study a BMI percentile equal to or greater than the 85^th ^percentile is referred to as overweight. Individual characteristics included in the model are gender (coded 0 = male and 1 = female) and the child's age at Cycle1 (coded 0 = age 2 and 1 = aged 3).

#### Family

A variable indicates if children are living in an 'intact family' with both biological parents. Previous research with the NLSCY has demonstrated that children living in an intact family have higher levels of health and well-being [24]. Children living in an intact family were coded as 1 and others as 0.

Income adequacy is a categorical measure of 1994 income based on the PMK report of household income during the previous 12 months [20]. The income categories are based on household income and household size. For example, a family of 2 would be classified in the highest group if making $60,000 CDN or more and a 3 person family would be classified in the highest group if making $80,000 CDN or more. This variable originally was created with 5 categories (lowest, low-middle, middle, high middle, and high). Lowest and low-middle were merged due to small sample sizes in the lowest group. Middle and high middle were combined to create three categories: low, middle (omitted reference group) and high.

Education of the PMK in 1994 was determined from a variable that groups education into 4 categories based on self-report: less than high school, high school, some postsecondary and postsecondary degree or diploma. Three analytic categories were created: less than high school, high school/some post secondary (omitted reference group), and post secondary degree/diploma.

#### Neighbourhood

Enumeration Areas, the smallest level of census geography in 1996 containing between 125 and 440 dwellings, were used as neighbourhood proxies. Neighbourhood low-income was assessed from the 1996 Census by calculating the proportion of the non-institutional population living below the low income cut-off. Proportions were divided into quintiles and the three middle quintiles were grouped resulting in three categories: 'least poor', 'middle' (omitted reference category) and 'most poor'.

Whether the neighbourhood was urban or rural (coded 0 = rural and 1 = urban) was determined by a variable indicating if it was in a Census Metropolitan Area (CMA). A CMA consists of one or more municipalities that form an urban core greater than 100,000 residents [25].

While data were available for 2,229 children at Cycle 5 some were excluded because of missing data for PMK education, family structure, or because a postal code could not be linked to neighbourhood data. There were 77 exclusions leaving a final sample of 2,152. Across all 5 time periods there are 8,915 valid BMI observations out of a total possible 10,760 (5 × 2152) so each child has an average of 4.14 out of 5 measurements. There was some variation in the number of valid BMI responses by family income: children from low income families have an average of 3.85 of 5 possible measures, middle income children have 4.19, and high income children have 4.30.

### Statistical analysis

Individual growth modelling techniques were used to analyze longitudinal data and the 5 waves of data available is adequate for modelling linear change [26]. A two-level model was specified in which observations at each time point (level 1) are clustered within individuals (level 2). The level-1 model describes within-individual change over time and the level-2 model assesses if predictor variables are related to inter-individual differences in change. An advantage of using this method is that children without complete BMI data for each survey cycle can be included [26].

A linear growth model was specified and the generic 2 level model used is:

BMI_it _= *π*_0i _+ *π*_1i _(Time_it_) + *ε*_it_

where BMI_it _refers to the outcome, BMI percentile for each child, i, at occasion t. Time_it _represents each measurement occasion for the ith child at time t and the initial measurement (Cycle 1, 1994) was assigned a value of zero. As such, *π*_0i _represents child i's true BMI percentile at Cycle 1 and *π*_1i _represents the slope of the change trajectory over a 2 year period for the *i*th child. Predictor variables were added as *β *coefficients on the intercept and change trajectory. Random effects were specified on the intercept *π*_0i _and slope *π*_1i _of the individual change trajectory with other coefficients entered as fixed effects. Iterative Generalized Least Squares was used for statistical estimation and final models used an unstructured co-variance matrix [27]. Sandwich estimators were used to calculate robust standard errors [27]. Following Singer and Willett [26] we used a sequential model building process beginning with a null or empty model and subsequently adding individual, family and neighbourhood variables. Six models are presented.

Descriptive statistics were computed using SAS^© ^and longitudinal models were computed using MLwin 2.02^© ^[27]. Variance estimates (not shown) of descriptive statistics were calculated using 1000 bootstrap weights and sampling weights were applied to longitudinal analysis [20].

## Results

**Figure 1:**
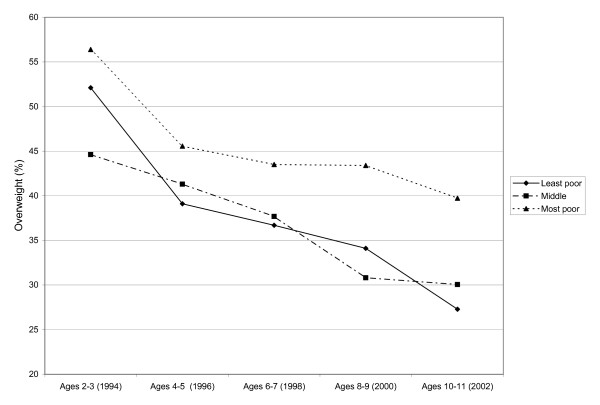
Prevalence of overweight among children in the NLSCY (aged 2–3, 1994) assessed bi-annually from 1994 to 2002 by neighbourhood income (%)*. *NLSCY = National Longitudinal Survey of Children and Youth (Canadian); Overweight based a Body Mass Index percentile ≥ 85^th ^using age and sex adjusted values from the US Center for Disease Control.

**Figure 2:**
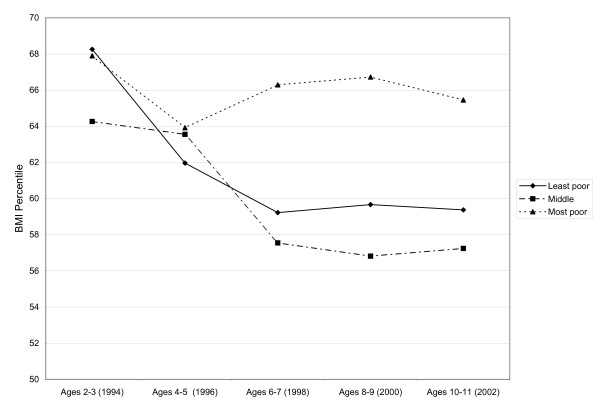
Average BMI percentile of children in the NLSCY (aged 2–3, 1994) assessed bi-annually from 1994 to 2002 by neighbourhood income*. * NLSCY = National Longitudinal Survey of Children and Youth (Canadian); BMI = Body Mass Index; BMI percentiles are age and sex adjusted values from the US Center for Disease Control values.

Table 1 shows the weighted descriptive statistics for the sample. Figure 1 shows that the 'most poor' neighbourhood had the highest percent overweight across all time periods while the 'middle' and 'least poor' neighbourhoods had values similar to each other. Figure 2 shows that the 'most poor' category had a higher BMI percentile than the 'middle' and 'least poor' and this disparity increases as children age.

**Table 1:** Descriptive statistics for children aged 2–3 in the NLSCY*

N = 2152	Percent (%)
**Baseline (1994)**	
**Individual Characteristics**	
Female	49.56
Male	50.44
Age = 2	48.52
Age = 3	51.48
**Income Adequacy in 1994**	
Low/low middle	20.28
Middle/high middle	64.43
High	15.29
**Parent Education in 1994**	
No high school certificate	14.98
High School/Some Postsecondary	46.41
Postsecondary degree	38.61
**Family Structure in 1994**	
Child living in an intact family	81.28
Child not living in an intact family	18.72
**Neighbourhood Low Income**	
Least Poor (less than 7.6%)	22.56
Middle (7.7 to 28.6%)	57.07
Most Poor (greater than 28.7%)	21.37
**Census Metropolitan Area**	
Urban	67.59
Rural	32.41
**Across Cycles (1994–2002)**	
**BMI Percentile (based on CDC values)**	Average (sd)
Ages 2–3 (1994)	65.77 (1.45)
Ages 4–5 (1996)	63.26 (1.29)
Ages 6–7 (1998)	59.85 (1.40)
Ages 8–9 (2000)	59.43 (1.54)
Ages 10–11 (2002)	59.57 (1.31)
**Overweight (BMI ≥ 85^th ^Percentile based on CDC values)**	Percent (%)
Ages 2–3 (1994)	48.71
Ages 4–5 (1996)	41.68
Ages 6–7 (1998)	38.67
Ages 8–9 (2000)	34.03
Ages 10–11 (2002)	31.53

* NLSCY = National Longitudinal Survey of Children and Youth (Canadian). Data used are for a cohort of children aged 2–3 at baseline (1994) and assessed bi-annually until 2002

Tables 2 and 3 present the results of the initial and full models and the *β*'s are the coefficient outcomes. Model A (Table 2) is an unconditional means model that has no predictors and is used to determine baseline estimates for comparison with subsequent models. The model goodness of fit statistic, the -2 log likelihood (88140.50), is compared with subsequent models to evaluate the model fit with the addition of predictor variables. Random effects are presented at the bottom of the table. Model B (Table 2) is an unconditional growth model which includes a single predictor of time. The fixed effects for this model include the intercept and change over time.

**Table 2:** Results for initial longitudinal models assessing BMI percentile change among children aged 2–3 in the NLSCY*

	Parameter	Model A		Model B	
		Estimate	95% CI	Estimate	95% CI
**Initial status**	*π*_0i_				
Intercept	*β*_00_	62.16	61.14 to 63.18	64.84	63.5 to 66.17
**Rate of change**	*π*_1i_				
Intercept	*β*_10_			-1.36	-1.83 to -0.89
Random Effects					
Level 2: In initial status	$\sigma_{0}^{2}$	119.60	106.66 to 132.53	124.72	105.03 to 144.42
Covariance	*σ*_01_			-1.80	-7.07 to 3.48
In rate of change	$\sigma_{1}^{2}$			0.94	-1.14 to 3.02
Level 1: Within person	$\sigma_{\varepsilon }^{2}$	975.73	946.37 to 1005.09	964.62	933.18 to 996.06
**Goodness of Fit**					
-2 Log Likelihood		88140.50		88102.66	

* NLSCY = National Longitudinal Survey of Children and Youth (Canadian); Estimates based on weighted data. Data used are for a cohort of children aged 2–3 at baseline (1994) and assessed bi-annually until 2002.

**Table 3:** Results for longitudinal models assessing the influence of neighbourhood and family characteristics on BMI percentile among children aged 2–3 in the NLSCY*

	Parameter	Model C		Model D	
		Estimate	95% CI	Estimate	95% CI
**Initial Status**	*π*_0i_				
Intercept	*β*_00_	71.05	67.1 to 74.99	70.58	65.82 to 75.34
Female	*β*_01_	-1.52	-3.53 to 0.49	1.82	-0.9 to 4.55
Age 2 in 1994	*β*_02_	-2.34	-4.35 to -0.33	-2.87	-5.6 to -0.13
Child living in an intact family, 1994	*β*_03_	-3.84	-7.06 to -0.62	-5.94	-10.24 to -1.64
Income adequacy: low/low middle, 1994	*β*_04_	0.17	-2.77 to 3.1	0.64	-3.42 to 4.71
Income adequacy: high, 1994	*β*_05_	0.31	-2.85 to 3.48	0.99	-3.21 to 5.19
No high school certificate (PMK), 1994	*β*_06_	2.44	-0.83 to 5.7	1.82	-2.75 to 6.39
Postsecondary degree (PMK), 1994	*β*_07_	-1.34	-3.55 to 0.88	-1.597	-4.62 to 1.42
Neighbourhood: least poor	*β*_08_	1.46	-1.02 to 3.95		
Neighbourhood: most poor	*β*_09_	4.03	1.2 to 6.86		
Neighbourhood: urban	*β*_010_	-3.51	-5.55 to -1.46		
**Rate of Change**	*π*_1i_				
Time	*β*_10_	-1.35	-1.82 to -0.88	-1.66	-3.31 to 0
Female	*β*_11_			-1.75	-2.69 to -0.82
Age 2 in 1994	*β*_12_			0.31	-0.63 to 1.25
Child living in an intact family, 1994	*β*_13_			1.17	-0.33 to 2.68
Income adequacy: low/low middle, 1994	*β*_14_			-0.02	-1.46 to 1.42
Income adequacy: high, 1994	*β*_15_			-0.72	-2.2 to 0.76
No high school certificate (PMK), 1994	*β*_16_			0.58	-1.01 to 2.16
Postsecondary degree (PMK), 1994	*β*_17_			0.082	-0.96 to 1.12
Neighbourhood: least poor	*β*_18_				
Neighbourhood: most poor	*β*_19_				
Neighbourhood: urban	*β*_110_				
**Random Effects**					
Level 2: Initial status	$\sigma_{0}^{2}$	120.12	100.51 to 139.74	120.6	101.18 to 140.01
Covariance	*σ*_01_	-2.12	-7.28 to 3.04	-1.36	-6.41 to 3.69
Rate of change	$\sigma_{1}^{2}$	0.95	-1.13 to 3.03	0.58	-1.46 to 2.61
Level 1: Within person	$\sigma_{\varepsilon }^{2}$	963.66	932.28 to 995.03	963.94	932.49 to 995.38
**Goodness of Fit**					
-2 Log Likelihood		88051.77		88048.71	

	Parameter	Model E		Model F	

		Estimate	95% CI	Estimate	95% CI
**Initial Status**	*π*_0i_				
Intercept	*β*_00_	66.71	63.84 to 69.57	72.43	67.31 to 77.55
Female	*β*_01_	1.96	-0.78 to 4.69	1.89	-0.83 to 4.61
Age 2 in 1994	*β*_02_	-2.84	-5.58 to -0.11	-2.91	-5.63 to -0.19
Child living in an intact family, 1994	*β*_03_			-6.42	-10.74 to -2.09
Income adequacy: low/low middle, 1994	*β*_04_			0.5	-3.58 to 4.59
Income adequacy: high, 1994	*β*_05_			1.68	-2.6 to 5.97
No high school certificate (PMK), 1994	*β*_06_			1.38	-3.21 to 5.97
Postsecondary degree (PMK), 1994	*β*_07_			-1.45	-4.45 to 1.56
Neighbourhood: least poor	*β*_08_	0.32	-3.08 to 3.72	0.87	-2.55 to 4.29
Neighbourhood: most poor		*β*_09_	2.15	-1.64 to 5.93	1.13	-2.69 to 4.95
Neighbourhood: urban	*β*_010_	-3.45	-6.2 to -0.69	-3.57	-6.38 to -0.76
**Rate of Change**	*π*_1i_				
Time	*β*_10_	-0.9	-1.89 to 0.1	-2.04	-3.79 to -0.29
Female	*β*_11_	-1.72	-2.65 to -0.78	-1.73	-2.66 to -0.8
Age 2 in 1994	*β*_12_	0.32	-0.63 to 1.26	0.3	-0.65 to 1.24
Child living in an intact family, 1994	*β*_13_			-1.31	-2.8 to 0.19
Income adequacy: low/low middle, 1994	*β*_14_			-0.19	-1.64 to 1.27
Income adequacy: high, 1994	*β*_15_			-0.69	-2.21 to 0.83
No high school certificate (PMK), 1994	*β*_16_			0.52	-1.06 to 2.11
Postsecondary degree (PMK), 1994	*β*_17_			0.05	-0.99 to 1.09
Neighbourhood: least poor	*β*_18_	0.32	-0.87 to 1.51	0.31	-0.89 to 1.51
Neighbourhood: most poor	*β*_19_	1.36	0.07 to 2.65	1.46	0.16 to 2.75
Neighbourhood: urban	*β*_110_	-0.12	-1.07 to 0.83	0.02	-0.96 to 0.99
**Random Effects**					
Level 2: Initial status	$\sigma_{0}^{2}$	123.83	104.03 to 143.63	119.51	100.05 to 138.97
Covariance	*σ*_01_	-2.04	-7.23 to 3.15	-1.41	-6.49 to 3.67
Rate of change	$\sigma_{1}^{2}$	0.65	-1.41 to 2.71	0.49	-1.54 to 2.52
Level 1: Within person	$\sigma_{\varepsilon }^{2}$	962.57	931.29 to 993.85	962.85	931.53 to 994.17
**Goodness of Fit**					
-2 Log Likelihood		88054		88028.2	

*NLSCY = National Longitudinal Survey of Children and Youth (Canadian); Estimates based on weighted data. Data used are for a cohort of children aged 2–3 at baseline (1994) and assessed bi-annually until 2002.

Model C (Table 3) presents the results of an initial status model that tests if predictor variables have an effect on children's initial status of BMI percentile and was used to gauge the effects of predictors without the addition of time. The estimate for age indicates that children aged 3 had a lower BMI percentile of -2.34 (95% CI -4.35 to -0.33) than children aged 2. Children in an intact family had an estimated differential in BMI percentile of -3.84 (95% CI -7.06 to -0.62) at initial status. The estimates for PMK education are in the expected direction but the confidence intervals include zero. Children living in the 'most poor' neighbourhood have an estimated differential in BMI percentile at initial status of 4.03 (95% CI 1.20 to 6.86) and children living in a CMA have an estimated BMI percentile difference of -3.51 (95% CI -5.55 to -1.46) at initial status. The -2 log likelihood for Model C was lower than Model B (88051.77 vs. 88102.66) indicating that the addition of predictor variables at initial status improves the model fit.

Model D (Table 3) includes effects of individual and family level predictors on both initial status and rate of change. Similar to the initial status model, being aged 3 and living in an intact family was associated with a decrease in BMI percentile at initial status. Effects over time show that being female was associated with a decreased rate of change.

Model E removes the family level variables and includes individual variables and neighbourhood income and urban/rural residence. This model shows the effect of neighbourhood income and urban/rural residence on BMI percentile change unadjusted for family factors. Living in a CMA decreased BMI percentile on initial status by -3.45 (95% CI -6.20 to -0.69) but the confidence interval for the estimate over time includes zero suggesting that there was no time varying effect. The initial estimate for the 'most poor' neighbourhood was 2.15 (95% CI -1.640 to 5.934) and the confidence interval included zero. The time varying estimate for the 'most poor' neighbourhood was 1.36 (95% CI 0.07 to 2.65) suggesting that neighbourhood income effects occur over time. When CMA residence was added as a time varying effect the initial status estimate decreased slightly to -3.45 (95% CI -6.20 to -0.69) and the confidence interval for the estimate over time included zero suggesting there was no time varying effect.

Model F includes both family and neighbourhood characteristics on initial status and rate of change. The -2 log likelihood was the lowest (88028.16) indicating this model best fits the data. While many of the family level variable estimates had confidence intervals that included zero they were retained for theoretical reasons and they contributed to the overall model fit [26]. Living in an intact family and living in a CMA was associated with a reduced BMI percentile at initial status. Living in the 'most poor' neighbourhood increased BMI percentile by 1.46 (95% CI 0.161 to 2.75) over time.

## Discussion

The contributions of this study are twofold. First, it contributes to research on neighbourhood effects on children's health by adopting a longitudinal perspective. Second, it contributes to our understanding of young children's trajectories of BMI by examining associations with both family and neighbourhood characteristics.

The principal finding of this paper is that the early neighbourhood environment influences children's BMI percentiles. Children living in the 'most poor' neighbourhoods have an increased rate of change in BMI percentile relative to children living in a 'middle' income neighbourhood. Living in the 'least poor' neighbourhood did not confer benefits suggesting that it is the effect of neighbourhood poverty rather than affluence that may matter most. The final model showed that over time living in the 'most poor' neighbourhood increases BMI percentile which is consistent with our hypothesis that neighbourhood characteristics may have a greater influence as children age. It is possible that neighbourhoods may become more important as children age and have more freedom to access the neighbourhood. It is also possible that disparities emerge over time as children are exposed to such environments over a longer time period.

A strength of this study is the availability of data for a nationally representative longitudinal sample of Canadian children allowing an examination of the influence of the early neighbourhood environment on BMI. A limitation is the use of PMK reported heights and weights which have well-known limitations compared to direct anthropological measures [28]. Evidence suggests parents underreport children's height leading to increased BMI values compared to direct measurements [29]. The decrease in BMI percentile over time is likely because even small underreports of height can considerably overestimate BMI in young children who are shorter. Another limitation is that parental BMI was not assessed in the NLSCY. A statistical limitation is that the few cases per neighbourhood prevented specifying a three level model.

During the 8 year follow-up children may have experienced changes in family income, parental education, family structure (divorce, remarriage etc) or may have moved neighbourhoods. Because we did not account for children who move neighbourhoods the model examines the influence of neighbourhood income at ages 2–3 on trajectories of BMI. Future research should examine the effects of moving and if children's trajectories differ based on the number of moves, timing of moves and whether moves are associated with changes in socioeconomic status. It is possible that moving to a neighbourhood with a different income profile could influence children's BMI trajectories. Over the 8 year period the percent of children in the lowest income category decreased from 19% to 7% and children in the highest category increased from 15% to 34%. Percent of children living in an intact family decreased from 81% to 69%. Accounting for these changes would have introduced significant model complexity.

Existing studies examining the influence of neighbourhood deprivation on overweight have been cross-sectional or have not included both family and neighbourhood variables [5,6,17,19]. These studies have found stronger effects for older children. Our study is the first we are aware of to examine longitudinal associations between the family and neighbourhood environment and children's body weight. A longitudinal study has found that socio-economic disparities in obesity did not increase as children aged from 11–16 [19]. The period from childhood to early adolescence may represent a critical period in which disparities in overweight by neighbourhood income are established. We did not find statistically significant results related to family income or PMK education reported in other Canadian studies [2,30].

The findings of this research suggest that obesity policies which focus on conditions of childhood including the places in which young children live may meet with the greatest success. Such policies may reduce the prevalence of obesity among all children and prevent the emergence of neighbourhood-based disparities in body weight as children age.

This study raises many questions for future research. This study found that children in rural areas have higher BMI percentiles than children in urban areas but the disparity, unlike neighbourhood income, does not increase with age. Future longitudinal studies examining urban-rural disparities in body weight are needed. This study did not evaluate the mechanisms and pathways through which low income neighbourhoods influence children's body weight over time. It is likely that differential access to food choices and opportunities for physical activity underlie this relationship. Cross-sectional studies have found that disadvantaged neighbourhoods have reduced access to healthy food options and parents report fewer safe parks and playgrounds [6,31,32]. However, longitudinal studies examining the influence of specific neighbourhood factors over time are needed to better understand how such factors influence children's bodyweight. At present we do not know if the influence of particular neighbourhood characteristics (e.g. parks and playgrounds, traffic) are greatest during certain periods in childhood and adolescence. This type of research may lead to the development of policies aimed at reducing neighbourhood disparities in overweight. Future research with a longer follow-up should investigate the effects of neighbourhood income on BMI from early adolescence to young adulthood.

## Conclusion

In conclusion, cross-sectional studies have demonstrated that neighbourhood disadvantage is associated with increased risk of overweight among children and youth. This study is the first we are aware of that uses longitudinal data to demonstrate that the disparity in BMI by neighbourhood income emerges between childhood and early adolescence. As obesity in childhood often persists into adulthood the findings of this study suggest that policies to prevent neighbourhood disparities in overweight should focus on young children.

## Competing interests

The author(s) declare that they have no competing interests.

## Authors' contributions

LO conceived of the idea for this project, conducted the analysis and prepared the manuscript. MH assisted with conceptualizing the paper and preparing the manuscript.

## Pre-publication history

The pre-publication history for this paper can be accessed here:


